# 5-Aminosalicylates Reduce the Risk of Colorectal Neoplasia in Patients with Ulcerative Colitis: An Updated Meta-Analysis

**DOI:** 10.1371/journal.pone.0094208

**Published:** 2014-04-07

**Authors:** Li-Na Zhao, Jie-Yao Li, Tao Yu, Guang-Cheng Chen, Yu-Hong Yuan, Qi-Kui Chen

**Affiliations:** Department of Gastroenterology, Sun Yat-Sen Memorial Hospital, Sun Yat-Sen University, Guangzhou, Guangdong, People's Republic of China; CWRU/UH Digestive Health Institute, United States of America

## Abstract

**Background:**

Although the chemopreventive effect of 5-aminosalicylates on patients with ulcerative colitis has been extensively studied, the results remain controversial. This updated review included more recent studies and evaluated the effectiveness of 5-aminosalicylates use on colorectal neoplasia prevention in patients with ulcerative colitis.

**Methods:**

Up to July 2013, we searched Medline, Embase, Web of Science, Cochrane CENTRAL, and SinoMed of China for all relevant observational studies (case-control and cohort) about the effect of 5-aminosalicylates on the risk of colorectal neoplasia among patients with ulcerative colitis. The Newcastle-Ottawa Scale was used to assess the quality of studies. Adjusted odds ratios (ORs) were extracted from each study. A random-effects model was used to generate pooled ORs and 95% confidence intervals (95%CI). Publication bias and heterogeneity were assessed.

**Results:**

Seventeen studies containing 1,508 cases of colorectal neoplasia and a total of 20,193 subjects published from 1994 to 2012 were analyzed. 5-aminosalicylates use was associated with a reduced risk of colorectal neoplasia in patients with ulcerative colitis (OR 0.63; 95%CI 0.48–0.84). Pooled OR of a higher average daily dose of 5-aminosalicylates (sulfasalazine ≥ 2.0 g/d, mesalamine ≥ 1.2 g/d) was 0.51 [0.35–0.75]. Pooled OR of 5-aminosalicylates use in patients with extensive ulcerative colitis was 1.00 [0.53–1.89].

**Conclusion:**

Our pooled results indicated that 5-aminosalicylates use was associated with a reduced risk of colorectal neoplasia in patients with ulcerative colitis, especially in the cases with a higher average daily dose of 5-aminosalicylates use. However, the chemopreventive benefit of 5-aminosalicylates use in patients with extensive ulcerative colitis was limited.

## Introduction

Ulcerative colitis (UC) is associated with an increased risk of colorectal cancer (CRC). A recent meta-analysis encompassing 8 population-based cohort studies reported a 1.6% prevalence of CRC in patients with UC, <1.0% by 10 years, 0.4%–2.0% by 15 years, and 1.1%–5.3% by 20 years. The rate of CRC was 2.4-fold higher than that in the general population [1]. Because of the importance of prevention and early detection of CRC in patients with UC, they have been discussed in many studies. Colonoscopic surveillance at regular intervals with multiple biopsies is considered the most effective way to detect and manage CRC early in UC patients and has been recommended for patients with long-standing UC [2]–[3]. On the other hand, the effect of potential chemopreventive drugs, such as 5-aminosalicylates (5-ASA), thiopurines, and folic acid, on UC patients was also studied, but the results remain controversial.

5-ASA, a first-line agent for the treatment of mild to moderate UC, includes sulfasalazine and nonsulfasalazine (including mesalamine, balsalazide, and olsalazine). Since the meta-analysis by Velayos et al in 2005 demonstrated that 5-ASA could reduce the risk of CRC in patients with UC, this matter has been further discussed by a number of studies [4]–[9]. The recent meta-analysis based on population-based studies by Nguyen et al showed that 5-ASA was not effective to prevent CRC in patients with inflammatory bowel disease (IBD) [10]. However, a recent long-term population-based study by Jess et al did not show the increasing risk of CRC in patients with Crohn's disease (CD) [11]. Furthermore, chronic inflammation is presumed to be a key factor of CRC development in patients with IBD, but 5-ASA plays a limited role in inducing remission and maintenance of CD [12]. As a result, it is necessary to separately analyze the chemopreventive effect of 5-ASA in patients with UC.

The objective of this study is to identify and update the association between 5-ASA use and colorectal neoplasia (CRN), defined as low-grade dysplasia, high-grade dysplasia, and CRC, in patients with UC.

## Methods

### Search strategies

Up to July 2013, we searched Medline, Embase, Web of Science, Cochrane CENTRAL, and SinoMed of China for all relevant articles on the effect of 5-ASA use on the risk of CRN among patients with UC. Medical subject heading (MeSH) or key words used in the research included “Salicylazosulphapyridine”, or “Salicylazosulfapyridine”, or “Sulphasalazine”, or “Sulfasalazine”, or “Mesalazine”, or “Mesalamine”, or “5-aminosalicylic acid”, or “5-aminosalicylate”, or “Balsalazide”, or “Olsalazine”, with “colorectal cancer”, or “colon cancer”, or “dysplasia”, or “carcinoma”, or “neoplasia”, or “advanced neoplasia”, and “inflammatory bowel disease”, or “ulcerative colitis”. Reference lists of all included articles were scrutinized to disclose additional literature on this topic. All abstracts, review articles, commentaries, and book chapters were excluded. If an author published more than one articles using the same case series, we only used the article that reported the data with the largest number of cases and the most completed information.

### Study selection

Two authors (LNZ and TY) selected target studies following a Proposal for Reporting Meta-analyses of Observational Studies in Epidemiology (MOOSE) guidelines [13]. Observational studies were included if they: 1) were case-control or cohort studies; 2) evaluated and clearly defined exposure to 5-ASA (sulfasalazine, mesalamine, balsalazide, and olsalazine) in patients with UC or IBD as a whole; 3) reported CRN outcomes; 4) reported odds ratios (ORs) or relative risks (RRs) with 95% confidence intervals (95%CIs), or provided data for their calculation; 5) were fully published.

### Data extraction and Quality assessment

All data were abstracted onto a standard form and crosschecked by two reviewers independently. The data extracted from all the studies were as follows: the last names of first authors, years of publication, time periods of study, study designs, countries of origin, study settings (population-based or hospital-based), total numbers of cases in each group, distribution of IBD diagnosis (UC and CD), types of medications, durations, and average daily doses, outcomes reported, ORs, RRs or hazard ratios with and without adjustment for potential confounders, corresponding 95%CIs, and potential confounders used for adjustment. Disagreements were resolved by consensus including a third author.

The Newcastle-Ottawa Scale (NOS) was used to evaluate the quality of each study. This measure assesses aspects of methodology in observational studies related to study quality, including 8 items categorized into 3 major categories: selection (4 items, 1 star for each), comparability (1 item, up to two stars) and exposure/outcomes (3 items, 1 star for each) [14]. The ultimate score of 6 stars or more was regarded as high-quality.

### Statistical analysis

We quantified the association between 5-ASA and CRN by using the Dersimonian and Laird random-effects model. Because the incidence of CRN was relatively low, the OR mathematically approximated the RR. All reported summary estimates were from studies with adjusted data, unless otherwise reported. When ORs were reported separately for different doses, types of 5-ASA (sulfasalazine or nonsulfasalazine) and durations of exposure, an overall estimate was calculated using the published individual adjusted ORs for each subgroup.

The *Q* and *I* ^2^ statistics were used to test statistical heterogeneity among studies. For the *Q* statistic, a *P* value of less than 0.1 is considered representative of statistically significant heterogeneity. *I* ^2^ is the proportion of total variation contributed by between-study variation. An *I* ^2^ index of around 25% is considered to demonstrate low levels of heterogeneity, 50% medium, and 75% high. Sensitivity analyses by reestimating pooled OR with omitting each study in turn were conducted to investigate the influence of each individual study on the overall meta-analysis summary estimate. Furthermore, subgroup analyses based on study designs (case-control or cohort), study settings (population-based or hospital-based), geographical regions, diseases (UC or IBD), and quality of studies were also performed to clarify the source of heterogeneity. In addition, cumulative meta-analysis was conducted to evaluate the change of the effect estimates over time. In the cumulative meta-analysis, studies were chronologically ordered by year of publication, and the pooled ORs were obtained at the end of each year.

Publication bias and small study effects were assessed by Begg's test and Egger's test, with *P*<0.05 considered to show significant publication bias. STATA (Version 12.0; STATA Corporation, College Station, TX, US) was used for all analyses.

## Results

### Literature search

**Figure 1:**
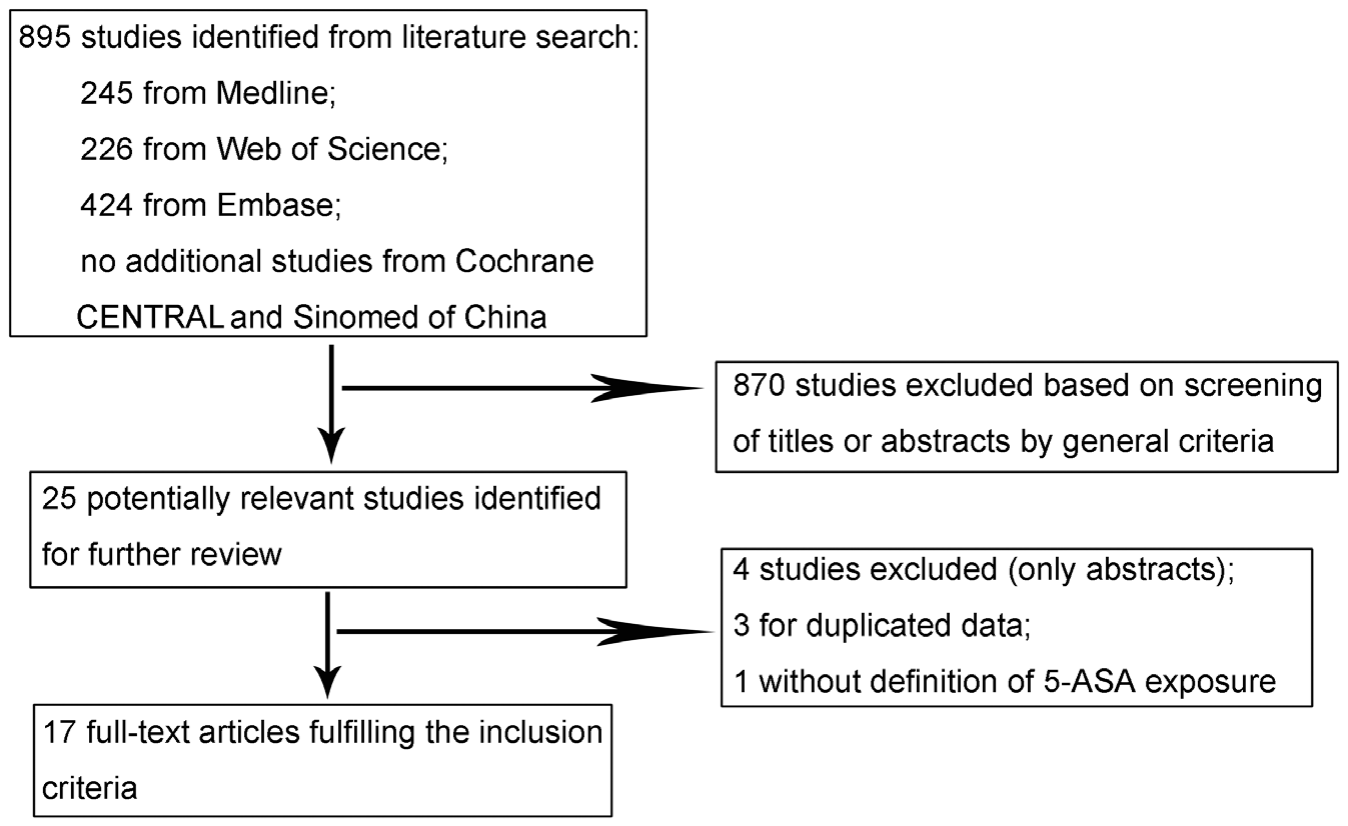
Flowchart of literature search for meta-analysis.

We reviewed 245 titles and abstracts from Medline, 226 from Web of Science, 424 from Embase, and no additional studies from Cochrane CENTRAL and SinoMed of China, and eventually chose 25 studies for further review [5]–[9], [11], [15]–[33]. Four supplementary studies were identified, but only their abstracts from conference proceeding of scientific meetings had been published [15]–[18]. They were excluded from the pooled analysis because of lack of details on key study variables. Three studies were excluded for their duplicated data [19]–[21]. One study was excluded without the definition of 5-ASA exposure [11]. Finally, 17 full-text articles were identified in this meta-analysis (Figure 1) [5]–[9], [22]–[33].

Seventeen studies containing 1,508 CRN cases, of which at least 75% were CRC cases, and a total of 20,193 subjects published from 1994 to 2012 were analyzed. Of the 17 studies, six were retrospective cohort studies and eleven case-control studies. Eight were population-based studies and nine hospital-based studies. Eight studies were conducted in Europe, seven in North America (six in USA, one in Canada), one in both Europe (Demark) and America, and one in Asia (China). Ten studies were exclusively based on UC patients; one study dealt with both UC and IBD patients; and six studies were based on IBD patients. The sample sizes ranged from 48 to 8,667 and the number of CRN cases varied from 10 to 364 (Table 1).

**Table 1:** Characteristics of studies of 5-ASA and colorectal neoplasia in patients with ulcerative colitis.

Study	Study period	Study design	Country	Study setting	Cases/controls	CD%&	CRN
Pinczowski, 1994	1965–1983	Case-control	Sweden	Population	102/196	0	CRC
Moody, 1996	1972–1992	Cohort	UK	Population	10/158	0	CRC
Lashner, 1997	1986–1992	Cohort	USA	Hospital	29/69	0	CRN
Eaden, 2000	1972–1989	Case-control	UK	Hospital	102/102	0	CRC
Lindberg, 2001	1973–1993	Cohort	Sweden	Hospital	50/93	0	CRN
Rutter, 2004	1988–2002	Case-control	UK	Hospital	68/136	0	CRN
van Staa, 2005	1987–2001	Case-control	UK	Population	100/600	15	CRC
Rubin, 2006	1985–2000	Case-control	USA	Hospital	26/96	0	CRN
Velayos, 2006	1976–2002	Case-control	USA	Hospital	188/188	0	CRC
Terdiman, 2007	2001–2003	Case-control	USA	Population	364/1172	NR	CRC
Jess, 2007	1940–2002	Case-control	USA+Denmark	Population	43/102	26	CRN
Gupta, 2007	1996–2007	Cohort	USA	Hospital	65/353	0	CRN
Tang, 2010	1970–2005	Case-control	USA	Hospital	18/30	17	CRC
Baars, 2011	1990–2006	Case-control	Netherlands	Population	173/393	34	CRC
Bernstein, 2011*	1995–2008	Cohort	Canada	Population	108/8559	41	CRC
Gong W, 2012	1998–2009	Case-control	China	Hospital	34/3888	0	CRC
van Schaik, 2012	2001–2009	Cohort	Netherlands	Population	28/2550	NR	AN

Note: CD: Crohn's disease, NR: not reported, CRN: colorectal neoplasia, CRC: colorectal cancer, AN: advanced neoplasia.

*With effect estimates for ulcerative colitis and inflammatory bowel disease.

& Percentage of CD in inflammatory bowel disease with CRN.

### 5-ASA and colorectal neoplasia

**Figure 2:**
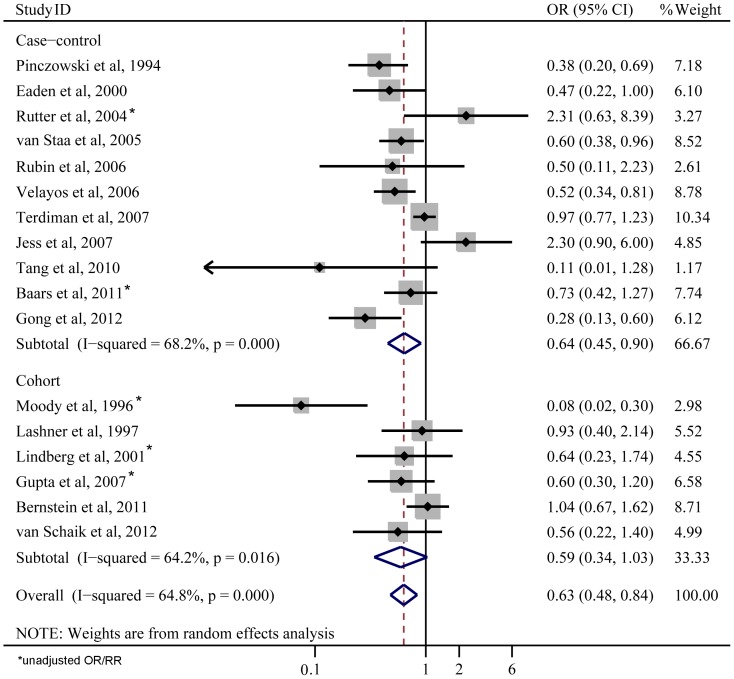
Forest plot (random-effects model) of 5-ASA use and colorectal neoplasia.

In a pooled analysis of all studies, 5-ASA use was associated with a reduced risk of CRN (OR 0.63; 95%CI 0.48–0.84; Figure 2). The protective effect remained significant with case-control studies (OR 0.64; 95%CI 0.45–0.90; Figure 2), and a trend towards reducing risk of CRN was also shown in retrospective cohort studies (OR 0.59; 95%CI 0.34–1.03; Figure 2). Although the pooled OR of cohort studies was lower than that of case-control studies, the range of 95%CI was wider and the difference was not statistically significant, indicating a lower power in cohort studies.

### Methodological quality and risk of bias

**Figure 3:**
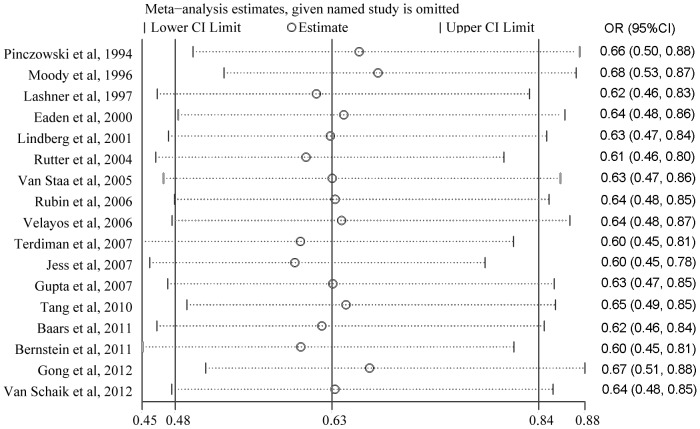
This diagram showed the influence of excluding each study in turn on the primary meta-analysis. The pooled ORs ranged from 0.60 to 0.68, with all showing statistically significant association between 5-ASA and colorectal neoplasia.

**Figure 4:**
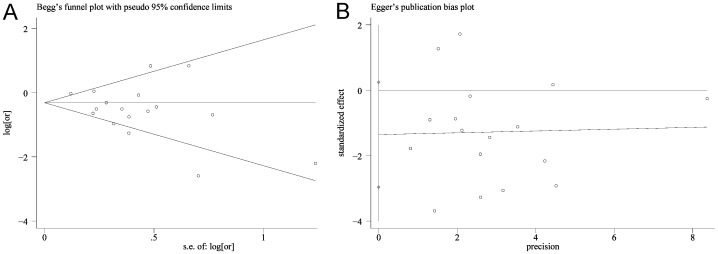
Begg's test and Egger's test. Begg's test and Egger's test identified no publication bias (Begg's test: Kendall's tau = −12, *P* = 0.65; Egger's test: bias = −1.36, *P* = 0.092).

There was significant heterogeneity across all studies (*I* ^2^ = 64.8%, *P*<0.001; Figure 2). Sensitivity analyses by reestimating pooled OR with excluding each study in turn were conducted. Pooled ORs ranged from 0.60 to 0.68, with all showing statistically significant association between 5-ASA and CRN (Figure 3). No obvious publication bias was found by Begg's test (Figure 4A) and Egger's test (Figure 4B).

### Subgroup analyses

#### Population-based and hospital-based

**Figure 5:**
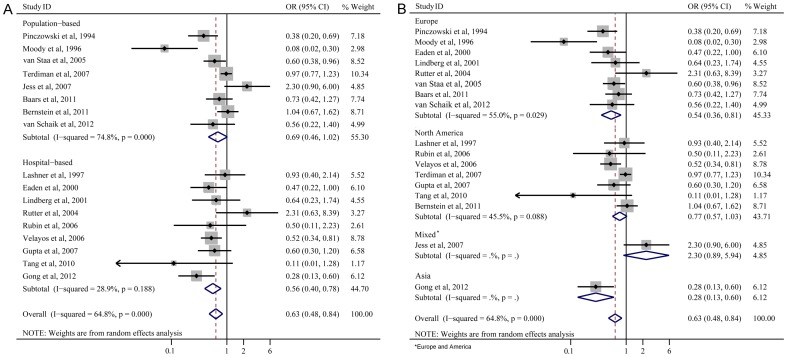
Forest plots of subanalyses of study settings (A) and geographical regions (B).

The protective effect of 5-ASA was significant in hospital-based studies (OR 0.56; 95%CI 0.40–0.78; Figure 5A), but not in population-based studies (OR 0.69; 95%CI 0.46–1.02; Figure 5A).

#### Geographical regions

The chemopreventive benefit was shown in Europe (OR 0.54; 95%CI 0.36–0.81; Figure 5B). However, no significant association was observed in North America (OR 0.77; 95%CI 0.57–1.03; Figure 5B). The OR of the study which included mixed patients in Europe (Demark) and America was 2.30 (95%CI 0.90–6.00). The OR of the study in Asia (China) was 0.28 (95%CI 0.13–0.60).

#### UC and IBD

**Figure 6:**
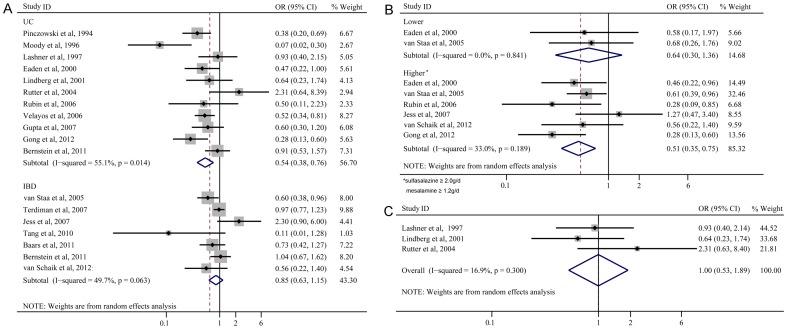
Forest plots of subanalyses of UC and IBD (A), average daily dose of 5-ASA use (B), and extensive UC (C).

5-ASA use was associated with a reduced risk of CRN (OR 0.54; 95%CI 0.38–0.76; Figure 6A) in UC patients. No significant association was shown in IBD patients (OR 0.85; 95%CI 0.63–1.15; Figure 6A).

#### Average daily dose of 5-ASA use

Six studies were included for their higher average daily dose of 5-ASA use (sulfasalazine ≥ 2.0 g/d, mesalamine ≥ 1.2 g/d). The use of a higher average daily dose of 5-ASA was associated with a lower risk of CRN (OR 0.51; 95%CI 0.35–0.75; Figure 6B). Only two studies defined a lower average daily dose of 5-ASA use (sulfasalazine <2.0 g/d, mesalamine<1.2 g/d) and the OR was 0.64 (95%CI 0.30–1.36; Figure 6B).

#### 5-ASA use in extensive UC

Three studies included patients with extensive UC (proximal to the splenic flexure). 5-ASA use in these patients was not associated with a lower risk of CRN (OR 1.00; 95%CI 0.53–1.89; Figure 6C).

### Cumulative meta-analysis

**Figure 7:**
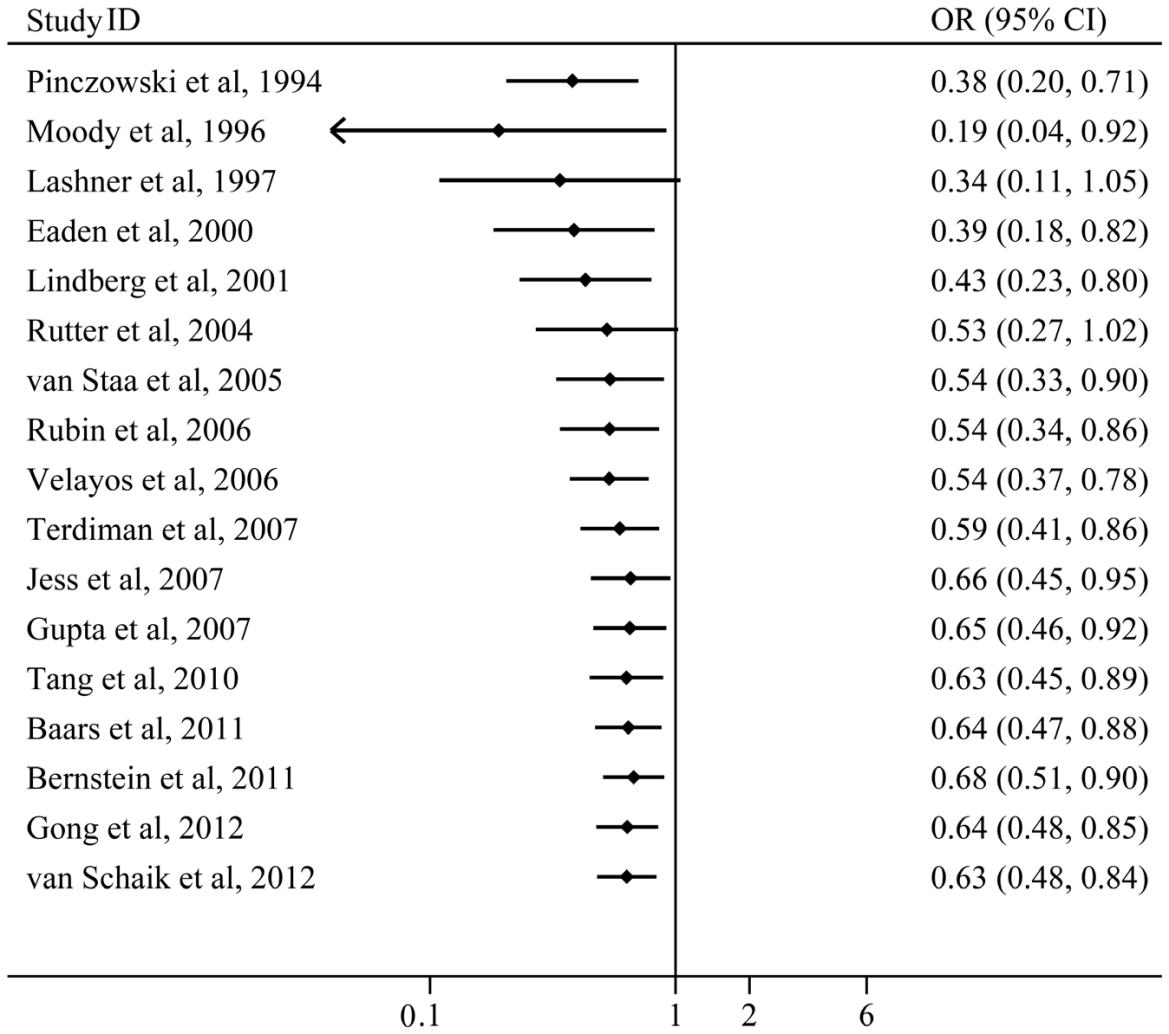
Forest plot of cumulative meta-analysis over time.

A cumulative meta-analysis of the total 17 studies was conducted to evaluate the cumulative effect estimates over time. In 1994, Pinczowski first reported that 5-ASA was a protective factor for CRC in UC patients (OR 0.38; 95%CI 0.20–0.69). Between 1994 and 2006, nine studies were published, with a cumulative OR of 0.54 (95%CI 0.37–0.78; Figure 7). Between 2007 and 2012, eight more publications were added cumulatively, resulting in an overall effect estimate of 0.63 (95%CI 0.48–0.84; Figure 7).

### Quality assessment and Subanalyses of high-quality studies

The results of the quality assessment according to NOS for case-control and cohort studies were shown in Table 2. The scores of the included studies ranged from four to eight stars. Twelve studies (71%) scored six or more were defined as high-quality, indicating a moderate to good study quality.

**Table 2:** Results of quality assessment by Newcastle-Ottawa Scale.

Study	1	2	3	4	5A	5B	6	7	8	scores
Case-control										
Pinczowski, 1994	☆	☆	☆	☆	☆	☆	−	☆	−	7
Eaden, 2000	☆	☆	−	☆	☆	☆	−	☆	−	6
Rutter, 2004	☆	−	−	☆	☆	☆	−	☆	−	5
van Staa, 2005	☆	☆	☆	☆	☆	☆	☆	☆	−	8
Rubin, 2006	☆	☆	−	☆	☆	☆	−	☆	−	6
Velayos, 2006	☆	☆	−	☆	☆	☆	☆	☆	−	7
Terdiman, 2007	☆	☆	☆	☆	☆	☆	☆	☆	−	8
Jess, 2007	☆	☆	☆	☆	☆	☆	☆	☆	−	8
Tang, 2010	☆	☆	−	☆	☆	☆	−	☆	−	6
Baars, 2011	☆	☆	☆	−	☆	☆	−	☆	−	6
Gong, 2012	☆	☆	−	☆	−	☆	−	☆	−	5
Cohort										
Moody, 1996	☆	☆	−	−	−	−	☆	☆	☆	5
Lashner, 1997	−	☆	−	−	☆	☆	☆	☆	☆	6
Lindberg, 2001	−	☆	−	−	−	☆	☆	☆	−	4
Gupta, 2007	☆	☆	−	−	−	−	☆	☆	☆	5
Bernstein, 2011	☆	☆	☆	−	☆	☆	☆	☆	☆	8
van Schaik, 2012	☆	☆	☆	−	☆	☆	☆	☆	☆	8

Note: For case-control studies, 1 indicates adequate definition of cases; 2, cases are representative of population; 3, community controls; 4, controls have no history of colorectal neoplasia; 5A, study controls for age and gender; 5B, study controls for additional factor(s); 6, ascertainment of exposure by blinded interview or record; 7, same method of ascertainment used for cases and controls; and 8, nonresponse rate the same for cases and controls. For cohort studies, 1 indicates exposed cohort truly representative; 2, nonexposed cohort drawn from the same community; 3, ascertainment of exposure; 4, outcome of interest not present at start; 5A, cohorts comparable on basis of age and gender; 5B, cohorts comparable on other factor(s); 6, quality of outcome assessment; 7, follow-up long enough for outcomes to occur (at least 1 year); and 8, complete accounting for cohorts (>75% follow-up or description provided of those lost).

The chemopreventive benefit was shown in high-quality studies (OR 0.70; 95%CI 0.54–0.92; *I* ^2^ = 55.7%; Table 3). Significant association was also observed in hospital-based, Europe, and UC studies, but not in population-based, North America, and IBD studies (Table 3).

**Table 3:** Subanalyses of high-quality studies.

	Number of studies	OR	95%CI	*I* ^2^
Total	12	0.70	0.54–0.92	55.7%
Study setting				
Population-based	7	0.79	0.57–1.08	62.5%
Hospital-based	5	0.54	0.39–0.76	0
Region				
Europe	6	0.55	0.43–0.69	0
North America	5	0.96	0.78–1.17	0
Disease				
UC	6	0.59	0.43–0.79	18.8%
IBD	6	0.85	0.63–1.15	49.7%

Note: UC: ulcerative colitis, IBD: inflammatory bowel disease.

## Discussion

Our pooled results from 17 studies indicated that the use of 5-ASA was associated with a reduced risk of CRN in patients with UC. The results also suggested that a higher average daily dose of 5-ASA use was more effective. The statistical analysis showed that there was significant heterogeneity across all studies (*I* ^2^ = 64.8%, *P*<0.001; Figure 2). Therefore, sensitivity analyses by reestimating pooled OR with omitting each study in turn were conducted to investigate the influence of each individual study on the overall meta-analysis summary estimate. The results indicated that pooled ORs ranged from 0.60 to 0.68, with all showing statistically significant association between 5-ASA and CRN (Figure 3). The cumulative meta-analysis over time showed that the estimates gradually became consistent, and the corresponding CIs narrowed down with the increase in the number of included studies ordered by year of publication. In addition, the chemopreventive benefit remained significant in high-quality studies. Meanwhile, there was no obvious publication bias found by Begg's test and Egger's test (Figure 4). These analyses enhanced the reliability of this meta-analysis.

In general, hospital-based studies were more susceptible to selection bias than population-based studies. Our results demonstrated that population-based studies did not show a significantly protective effect on reducing the risk of CRN, while hospital-based studies did. This difference could be elucidated by the following two reasons. Firstly, hospital-based studies are prone to selecting more severe cases and have a higher risk of CRC compared with population-based studies. In addition, some population-based studies reported that patients with UC only had a modestly increasing risk of CRC, thus the benefit of 5-ASA for prevention of CRC in population-based studies might be inconspicuous [34]–[36]. Secondly, the compliance of the selected population in hospital-based studies was different from the general population, like avoiding unhealthy life style and taking 5-ASA more regularly. However, because of the significant heterogeneity among the population-based studies (*I* ^2^ = 74.8%, *P*<0.001; Figure 5A), this result requires further investigations.

We found that the risk of CRN in Europe was significantly reduced, but it was not the case in North America. The reason for the difference is unclear. The differences in genetic susceptibility, culture, and lifestyle may explain part of the inconsistency of the results. Other confounders, such as the colectomy rates, the severity and extent of UC, and the selected population of UC and IBD, might also play an important role in the discrepancy.

The risk factors of CRC in patients with UC include: 1) duration/early onset age, severity and extent of UC; 2) degree of histological/endoscopic inflammatory activity; 3) family history; 4) primary sclerosing cholangitis [12]. The first two risk factors are related to inflammatory burden. And chronic mucosal inflammation is a putative predominant mechanism responsible for increased risk of CRC. 5-ASA, as an effective anti-inflammation drug for patients with UC, is reasonably considered as a chemopreventive drug for CRC. Furthermore, the molecular mechanisms of anticancer effects of 5-ASA are plausible. 5-ASA may inhibit cell-cycle progression by interfering with TGF-β, TNF-α, NF-κB pathway and Wnt/β-catenin signaling, improve DNA replication fidelity, and reduce free radicals as a scavenger of reactive oxygen species [37]. These support our findings that 5-ASA use could reduce the risk of CRN in patients with UC.

Consistent with the meta-analysis conducted by Velayos et al, the six studies with a higher average daily dose of 5-ASA (sulfasalazine ≥ 2.0 g/d, mesalamine ≥ 1.2 g/d) in our study also suggested a chemopreventive effect in patients with UC [4]. In our study, all of the three studies including patients with long-term and extensive UC illustrated that 5-ASA use was ineffective on CRN prevention [30], [32], [33]. Pooled OR of this subanalysis was 1.00 (95%CI 0.53–1.89; Figure 6C). One possible explanation is that the management and maintenance of remission of extensive UC is more difficult than that with distal colitis by 5-ASA use. Other long-term maintenance drugs (immunomodulators, like thiopurine and azathioprine, or biologics) are recommended to patients with extensive UC who have failed 5-ASA therapy, allowing such patients without 5-ASA use in quiescent disease [41]. Besides, it has been demonstrated that thiopurines can protect IBD patients against advanced neoplasia [27]. Accordingly, immunomodulators or biologics use in patients with extensive UC may exert significant bias on the effect of 5-ASA use in CRN prevention. Among the three studies, it was suggested that azathioprine showed no significant protective effect against CRN by Lasher and Rutter, but immunomodulators use was not reported by Linderberg [30], [32], [33]. Additionally, adherence and average daily dose of 5-ASA use, which have considerable influence on the effect of 5-ASA use on long-term maintenance therapy in patients with extensive UC, were not reported in all of the three studies [42]. Therefore, this result should be carefully interpreted and needs further investigations.

The included IBD studies (including CD patients) might result in a significant bias on the chemopreventive effect of 5-ASA on UC patients, because according to a recent Cochrane Database review and clinical guidelines, 5-ASA is not an effective drug to control CD and not highly recommended for the management of CD [38]–[40]. However, we included the six IBD studies and mixed them with the UC studies as the main analysis, considering that the six IBD studies were recently well-designed ones with large database and a majority of the subjects in these studies had UC. Nevertheless, we conducted a subgroup analysis of UC and IBD, demonstrating that the use of 5-ASA was associated with a reduced risk of CRN (OR 0.54; 95%CI 0.38–0.76; Figure 6A) in UC patients, but no significant association was shown in IBD patients (OR 0.85; 95%CI 0.63–1.15; Figure 6A).

There were several limitations in our study. Firstly, studies included in this meta-analysis were all observational studies (case-control and retrospective cohort studies). Therefore, the influence of confounding variables was inevitable in such long period studies. The main confounders, such as the colectomy rates and the severity and extent of UC, could not be obtained from all studies [8]. Secondly, most of these studies extracted data of 5-ASA exposure from medical records and might result in inaccuracy. Apart from that, most of them did not describe clearly the duration and average daily dose of 5-ASA use. Thirdly, there was significant heterogeneity among all the studies. Although we investigated the influence of each individual study on the overall estimate and conducted subgroup analyses according to different study settings, geographical regions, and average daily doses of 5-ASA use, the heterogeneity remained significant in some of the subgroup analyses and unable to be clearly classified.

In summary, our pooled results indicated that 5-ASA use was associated with a reduced risk of CRN in patients with UC, especially in the cases with a higher daily dose of 5-ASA use. However, the benefit of 5-ASA use in preventing CRN in patients with extensive UC was limited. Large population-based and well-designed studies are needed to confirm these results.

## Supporting Information

Checklist S1 **PRISMA Checklist.**
(DOC)
